# Medical Wards

**Published:** 1871-12

**Authors:** 


### MEDICAL WARDS.

Services of PROF. N. S. DAVIS.

CASE I. BRONCHO-PNEUMONIA, WITH TYPHOID SYMPTOMS.—This patient is in the second week of typhoid fever with a pulmonary complication which makes his condition quite serious. The expectoration has been tinged with blood for two or three days, and there is dullness over a part of one lung with severe bronchial cough. These symptoms led to the conclusion that the inflammatory action, which had existed in the capillary bronchial tubes was extending to some of the lobules of the lungs. The typhoid depression was considerable and the indications were to sustain the circulation and innervation, and to induce freer secretion from the pulmonary mucous membranes, and thus lessen the tumefaction and admit a larger supply of oxygen to the blood. The first indication is well met by the following prescription:

℞ Ammonia carbonate, gr. jv.
Quinia sulph, . . gr. ij.
Camphor gum, . . gr. j.

Take every four hours.

To accomplish the second object the mixture of muriate of ammonia, tartrate of antimony and morphia in syrup of liquorice was given every four hours, alternating with the other medicine. A blister was also applied to the sternum. At present the pulse is fuller and less frequent, the expectoration more abundant, and the whole aspect of the case decidedly better.

NOTE.—At a clinic four days later the attention of the class was again called to this patient; the pulmonary symptoms had nearly disappeared, and the fever was better. The treatment had not been changed.

CASE II. TYPHOID FEVER WITH CAPILLARY BRONCHITIS.—This is a case of typhoid fever complicated with capillary bronchitis, which has been on the use of the emulsion of turpentine and laudanum, and the muriate of ammonia mixture. The former medicine has brought the bowels into a satisfactory condition, but the want of innervation has been so great that the patient was in much danger from the depression of the nerve centers. To remedy this he was given the following prescription :

| | | |
|---|---|---|
| ℞ | Strychnia, . | gr. j. |
| | Acid, nitric, | ʒj. |
| | Tinct. opii, . | ʒiij. |
| M | Aquæ, . . | ℥jv. |

DOSE—One tea-spoonful in sweetened water every four hours.

Now, after two days, the patient is improved, the countenance looking better and the mind clearer ; but the typhoid depression is still considerable, and it is best that the tonic be continued. The muriate of ammonia mixture is also to be continued on account of the bronchitis. On examining the chest the spaces above the clavicles and between the ribs are seen to sink at the beginning of each inspiratory act and to bulge during expiration. This is evidently due to the difficulty in getting air into the air cells on account of the obstruction of the capillary tubes ; on the expansion of the chest the air cannot enter fast enough to fill the cells and a partial vacuum is produced ; atmospheric pressure from without then presses in the soft tissues at the intercostal spaces ; while in expiration the air is with equal difficulty passed out of the cells through the obstructed tubes. Thus forcible inspiration and slow expiration is the characteristic respiration where the air cells are themselves permeable but the capillary tubes partially closed. But when the air cells are infiltrated the respiratory acts are short.

Sometimes when there is typhoid depression, wandering of the mind and bronchial tightness, chloroform is useful, and in this case may be given with propriety according te the following formula.

| | | |
|---|---|---|
| ℞ | Chloroform, | ʒiij. |
| | Accaciæ, . | ʒvj. |
| | Saccchari, . | ʒvj. |
| M | Aquæ, . . | ℥vj. |

DOSE—A table-spoonful ever four hours, alternately with the other medicine.

